# Genome Sequences of Rhinovirus B Isolates from Wisconsin Pediatric Respiratory Studies

**DOI:** 10.1128/genomeA.00202-14

**Published:** 2014-03-27

**Authors:** Stephen B. Liggett, Yury A. Bochkov, Tressa Pappas, Robert F. Lemanske, James E. Gern, Naomi Sengamalay, Xuechu Zhao, Qi Su, Claire M. Fraser, Ann C. Palmenberg

**Affiliations:** a Department of Medicine, University of South Florida Morsani College of Medicine, Tampa, Florida, USA; b Department of Pediatrics, University of Wisconsin School of Medicine and Public Health, Madison, Wisconsin, USA; c Institute for Genome Sciences, University of Maryland School of Medicine, Baltimore, Maryland, USA; d Institute for Molecular Virology, University of Wisconsin-Madison, Madison, Wisconsin, USA

## Abstract

Nearly full-length RNA genome sequences for 39 rhinovirus B isolates (RV-B), representing 13 different genotypes, were resolved as part of ongoing studies at the University of Wisconsin that attempt to link rhinovirus (RV) diversity and respiratory disease in infants.

## GENOME ANNOUNCEMENT

The University of Wisconsin hospitals and clinics in Madison, WI, support several ongoing studies examining why some children have mild illnesses while others have severe colds or even asthma attacks when they catch a rhinovirus-induced cold. The Childhood Origins of Asthma (COAST), RhinoGen, and T Regulatory Cells and Childhood Asthma (T-Reg) studies screen cohort infant nasal secretions from well/sick visits, with the goal of correlating respiratory viruses with the severity of illness. Of samples collected between 1999 and 2010, hundreds were identified as solitary infections by RNA rhinoviruses (RV), organisms in the *Enterovirus* genus of the *Picornaviridae* family. The screens used multiplex PCR assays (1), rhinovirus PCR (2), or both. The initial partial sequencing assigned species (A, B, or C) and preliminary genotype identifications, as has been reported (3). Multiple isolates were then reexamined using massively parallel sequencing techniques. The methods (4) were applied directly to patient samples without viral propagation in cells, a procedure that automated the sequence derivations and eliminated variant selection bias that might have been introduced by amplification. The single-pass methodology gave, on average, 93% full-genome coverage to a depth of 8 to 10 reads for 179 study-specific isolates. The sample titers that contributed to successful datasets ranged from 6 × 10^2^ to 1.6 × 10^8^ virions (viral RNA [vRNA] copy number per 350 µl of nasal sample, determined by quantitative real-time PCR [qRT-PCR]), with a median value of ~6 × 10^4^ virions.

In historic RV taxonomy, panels of clinical isolates, as archived by the American Type Culture Collection, were indexed into an initial 99 RV-A and RV-B types after an assessment of antigenic cross-reactivity in rabbits (5). Current classification schemes assign RV strains to one of 3 species (A, B, or C) if they share >70% amino acid identity in the P1, 2C, and 3CD regions with other known members. Within species, the isolates are subdivided into numeric genotypes that respect the historic serotype-naming system but now rely almost entirely on observed pairwise nucleotide identity (>87 to 88%) in the VP1 or VP4/VP2 coding sequences (6). The delineations and potential new genotypes are periodically reviewed by the *Picornaviridae* Study Group of the International Committee on Taxonomy of Viruses (ICTV). The preferred nomenclature (6) designates the species letter (A, B, or C) and type number (e.g., A16). The isolate designations are unique to each accession number.

Within this context, the Wisconsin study resolved nearly full-genomes for 39 RV-B isolates representing 13 different genotypes. The data add depth to observations for the new genotypes B101 and B102 (7) and define novel genotypes, B103 and B104. As a consequence, a previous B52 field isolate (accession no. FJ445188) was also reclassified as B104 (3). Relative to prototype RV-B genomes, which average ~7,209 bases (b) (8), most of the 39 new assemblies were missing the difficult-to-sequence 5′ and/or 3′ termini (average, Δ404 b) and occasionally, short internal fragments (<100 b) for which the contigs were not explicitly linked. Nevertheless, every derived sequence (average, 6,753 b; median, 6,806 b) was unambiguously aligned with the index RV compilation (8) for accurate type identification (6).

### Nucleotide sequence accession numbers.

Each contiguous RV-B data set has been deposited in GenBank using the listed accession numbers. Each unit described here is the first genome version of the sequence of that isolate: B04, JN798573; B06, JN562723, JN815243, JQ747745, JQ747748, and JX193795; B42, JF781498, JF781507, and JN562724; B48, JN990698; B69. JN562721 and JQ245970; B70, JN990706, JQ245974, and JX074054; B72, JN562726, JN614997, JN798562, and JQ245969; B83, JN990701; B84, JF781499, JF781502, JN541271, JN614991, JN798588, JQ837723, and JX074048; B101, JF781500, JF781501, and JX074052; B102, JX074053; B103, JN614996, JN798572, JN815239, JQ245972, JQ837717, JQ837721, and JQ994497; B104, JF781506.
